# Increased interferon I signaling, DNA damage response and evidence of T-cell exhaustion in a patient with combined interferonopathy (Aicardi-Goutières Syndrome, AGS) and cohesinopathy (Cornelia de Lange Syndrome, CdLS)

**DOI:** 10.1186/s12969-024-01050-7

**Published:** 2025-01-27

**Authors:** Sorina Boiu, Nikolaos Paschalidis, George Sentis, Theodora Manolakou, Andrianos Nezos, Manolis Gialitakis, Maria Grigoriou, Erato Atsali, Melpomeni Giorgi, Argirios Ntinopoulos, Clio Mavragani, Periklis Makrythanasis, Dimitrios T. Boumpas, Aggelos Banos

**Affiliations:** 1 Third Department of Pediatrics, Pediatric Rheumatology Unit, National and Kapodistrian University of Athens, ‘Attikon’ General University Hospital, Athens, Greece; 2 Department for Community Health, Faculty of Science and Medicine, University of Fribourg, Fribourg, Switzerland; 3 Department of Pediatrics, Fribourg Hospital, Fribourg, Switzerland; 4 Laboratory of Immunogenetics of Pediatric Autoimmune Diseases, Imagine Institute, INSERM UMR 1163, Paris, France; 5 Biomedical Research Foundation, Academy of Athens, Athens, Greece; 6 Laboratory of Autoimmunity and Inflammation, Center for Clinical, Biomedical Research Foundation, Experimental Surgery and Translational Research, Academy of Athens, Athens, Greece; 7 Department of Physiology, National and Kapodistrian University of Athens Medical School, Athens, Greece; 8 Third Department of Pediatrics, Attikon University Hospital, National and Kapodistrian University of Athens, Athens, Greece; 9 Third Department of Pediatrics, Pediatric Neurology Unit, National and Kapodistrian University of Athens, ‘Attikon’ General University Hospital, Athens, Greece; 10 Laboratory of Medical Genetics, Medical School, National and Kapodistrian University of Athens, Athens, Greece; 11 Department of Genetic Medicine and Development, Medical School, University of Geneva, Geneva, Switzerland; 12 4th Department of Internal Medicine, National and Kapodistrian University of Athens, Athens, Greece

**Keywords:** Lupus, Interferonopathy, Cohesinopathy, DNA damage response, Aicardi-Goutiéres Syndrome, Cornelia de Lange Syndrome

## Abstract

**Background:**

Type I interferonopathies including Aicardi-Goutiéres Syndrome (AGS) represent a heterogeneous group of clinical phenotypes. Herein, we present a Case with combined AGS and Cornelia de Lange Syndrome (CdLS)—a cohesinopathy—with comprehensive analysis of the immune and genomic abnormalities.

**Case and methods:**

A 20-year old man presented with chilblain lesions and resorption of distal phalanges of fingers and toes, somatic and psychomotor retardation, microcephaly, synophrys, hearing losing and other aberrancies consistent with the phenotype of CdLS. We used whole exome sequencing to genetically map the associated mutations and performed transcriptome profiling and enrichment analysis in CD14^+^ monocytes of the patient and immune phenotyping by mass cytometry (CyToF), comparing to healthy individuals and lupus patients as disease controls. DNA damage response was assayed by confocal microscopy in the peripheral blood of this patient.

**Results:**

Next generation exome sequencing confirmed a homozygous SAMHD1 gene mutation and a hemizygous non-synonymous mutation on SMC1A gene, responsible for the AGS and CdLS, respectively. Transcriptome profiling of CD14^+^ monocytes of the patient showed enrichment of type I IFN signaling and enhanced DNA damage response pathway. Broad immune phenotype of the peripheral blood of the patient revealed absence of activated T cell populations, increased frequency of NK cells and plasmablasts and enhanced granulocytic lineage. Further analysis suggested activation of the ATM branch of DNA damage response and increased apoptosis in the periphery of the patient.

**Conclusions:**

A rare case of a patient bearing two genetic lesions (responsible for AGS/CdLS syndromes) exhibits distinctive features of genomic damage and interferon responses. Immune phenotype revealed granulocytic skewing and absence of activated T cells compatible with chronic antigenic stimulation and/or homing of these cells at sites of inflammation.

**Supplementary Information:**

The online version contains supplementary material available at 10.1186/s12969-024-01050-7.

## Introduction

Autoimmune phenotypes driven by monogenic mutations are rare, though recently their diagnosis and study are supported by next generation sequencing technologies. Inborn errors of immunity (IEIs) can result to various immune-related phenotypes, with autoimmunity, autoinflammation and immunodeficiency being the most outstanding among them.

Aicardi-Goutières Syndrome (AGS), the prototype of the type I interferonopathies, is a genetically determined encephalopathy caused by mutations in any one of ten distinct genes (ADAR, IFIH1, RNASEH2A, RNASEH2B, RNASEH2C, SAMHD1, TREX1, DNASE2, LSM11 and RNU7-1) [1, 2]. Mutations in these genes affect the sensing and/or metabolism of nucleic acids, triggering an autoimmune response with an increase in interferon-α (IFN-α) production. AGS shares common characteristics with both congenital infection sequelae and systemic lupus erythematosus (SLE). Patients can demonstrate heterogeneous phenotypes, while beyond the central nervous system, other organs, such as the skin (mainly chilblain lesions), thyroid, eyes and blood vessels, can be involved with high variability [3].

Cornelia de Lange syndrome (CdLS) is a member of a class of developmental disorders referred to as cohesinopathies, which result from mutations in any of seven genes encoding subunits or regulators of the cohesin complex (*NIPBL, SMC1A, SMC3, RAD21, HDAC8 SMC1A*, *SMC3*, *RAD21*, *BRD4*, *HDAC8* and *ANKRD11)* [4]*.* Cohesin complex and its accessory protein members facilitate sister chromatid cohesion, DNA damage repair, genome compartmentalization and transcriptional regulation [5]. CdLS patients have variable phenotypes, characterized by facial dysmorphia, hypertrichosis, upper limb malformations, growth and cognitive retardation among numerous other signs and symptoms [4]*.*

Rare genetic syndromes were underdiagnosed until recently, as not all clinical cases exhibit the same level of severity with milder cases being increasingly recognized after broader utilization of next generation technologies, specifically whole exome or whole genome sequencing (WES/WGS). Advances in transcriptome sequencing, mass cytometry and single-cell studies have enabled the characterization of the aberrancies presented with these diseases. From a therapeutic standpoint, extreme phenotypes of interferonopathies may respond to Janus Kinase Inhibitors (such as tofacitinib and ruxolitinib), however clinical experience with these agents is limited to case reports[6–8]. Encouraging initial results have also been observed with inhibition of reverse transcriptase [9]. Importantly, immune- or autoimmune related symptoms are alleviated, but neurological manifestations usually do not abate. Cohesinopathies are characterized by lack of effective drug therapy, except of experimental trials targeting the mTOR pathway [10].

In this study, we describe an extreme genetic phenotype of two simultaneous genetic lesions, leading to broad alterations in immunophenotype and transcriptome. DNA damage response and type I interferon signaling are the key pathways likely driving the disease manifestations in our Case.

## Materials and methods

### DNA extraction

DNA extraction was performed for the patient’s sample with Trizol Reagent (Ambion, Life Sciences, USA) according to the manufacturer’s instructions. The quantity and quality of the DNA sample was spectrophotometrically tested (Biospec Nano, Japan).

### Whole Exome Sequencing (WES)

WES libraries were prepared with the Twist library preparation EF 2.0 protocol and the Twist Comprehensive Exome Panel, according to manufacturer’s instructions. Quality of the libraries was verified with Agilent Bioanalyzer DNA1000 and HS chips and quantify with the qubit HS spectrophotometric method. WES libraries were sequenced in the Illumina NovaSeq 6000 sequencer. Bioinformatics analysis was performed using custom pipeline which utilizes published algorithms in a sequential manner (BWA for mapping the reads, SAMTools for processing of files, GATK for detection of variants, ANNOVAR for the annotation) [11–14].

### Interferon signature

Whole blood RNA was extracted from the patient and age and sex matched healthy controls (*n* = 4) with Trizol Reagent (Ambion, Life Sciences, USA) according to the manufacturer’s instructions. All samples were treated with DNAse I (Qiagen, Germany) prior to cDNA synthesis. The quantity and quality of RNA samples were spectrophotometrically tested (Biospec Nano, Japan). One μg of total RNA was reverse-transcribed using the Superscript III reverse transcriptase system from Invitrogen (Carlsbad, CA). Oligo-dT primer (0.5μM) (Qiagen, Germany) was used to amplify mRNA and RNAse inhibitor was included to prevent degradation (RNAseOUT, Invitrogen, USA). Quantitative Real-Time Polymerase Chain Reaction (qRT-PCR) was used to quantify specific cDNAs using the Bio-Rad IQ5 thermocycler and the IQ Bio-Rad SYBR Green Supermix (Bio-Rad, Hercules, CA). Briefly, genes preferentially induced by type I IFNs were selected and included: myxovirus (influenza virus) resistance 1 (MX-1), interferon-induced protein with tetratricopeptide repeats 1 (IFIT-1) and interferon- induced protein 44 (IFI44). As an internal control and normalization gene we used the glyceraldehyde phosphate dehydrogenase (GAPDH). Type I IFN score was calculated as the sum of the study subject’s relative expression for each of the three genes preferentially induced by type I IFN [15].

### Library construction

Peripheral CD14^+^ cells (isolation through Microbeads, Miltenyi Cat No 130–050–201) of patients and healthy individuals were total RNA-sequenced. Nucleospin RNA Kit (Macherey Nagel) was used for RNA isolation. Library construction was performed using NEBNext® rRNA Depletion Kit v2 (#E7400, New England Biolabs) and NEBNext® Ultra™ II Directional RNA Library Prep with Sample Purification Beads Kit (#E7765 New England Biolabs). Library quality was assessed using a 2100 Bioanalyzer (Agilent), and a Qubit 4 Fluorometer with dsDNA HS assay kit (#Q32854, Thermo Fisher Scientific) was used for quantitation of libraries. 75-bp paired-end sequencing was performed on an Illumina HiSeq2000 System.

### Sequencing QC and analysis

Sequencing data quality assessment was performed using FastQC software (version:0.11.9) [16]. Low-quality bases (Q < 30) of the 3′ end and adapter sequences were removed using Cutadapt (v:1.18) [17]. STAR (v:2.6.1b) [18] was used to align trimmed reads to the human reference genome (v:hg38) with GENCODE annotation (v:39) [19]. Bam files were sorted using Samtools (v:1.9) [20] and gene expression counts were generated using HTSeq (v:0.11.0) [21]. In general, RNA sequencing quality control and analysis was performed as previously described [22].

### Differential expression (DE) analysis

The edgeR [23] package (v:4.0.6) was used in R [24] (v:4.3.1) to normalize the raw counts and compare the gene expression between Case, SLE patients and healthy individuals. Genes were considered significantly differentially expressed when *P*-value < 0.05 and |FC|≥ 1.5. Package ggplot2 (v:3.4.4) [25] and ComplexHeatmap [26] were used to visualize the results.

### Gene set enrichment analysis

A ranked gene list was created for each DE comparison by ranking genes according to the product of log2(FoldChange) multiplied by -log10(*P*-value) in descending order. Preranked gene-set enrichment analysis (GSEA) was performed against Hallmark, Gene Ontology (C5:GO) and Canonical Pathways (C2:CP) collections of MSigDB [27] (v2023.2.Hs) using the GSEA [28] software (v:4.3.2).

### Gene set variation analysis

Gene set variation analysis (GSVA) was performed using the GSVA R package (v:1.50.0) [29] against genesets of MSigDB (v2023.2.Hs) related to Interferon signaling and DNA damage response. Another set comprised of interferon stimulated genes compiled from a previous publication [30] was also used along with the MSigDB genesets.

### Human Peripheral Blood Mononuclear Cell (PBMCs) isolation, in vitro culture & flow cytometry

Heparinized blood (10ml) was collected from healthy subjects and individual with AGS. PBMCs were isolated on Histopaque-1077 (Sigma #10,771) density gradient. Briefly, blood was diluted 1:1 with phosphate-buffered saline (PBS) and layered over Histopaque-1077 solution. Samples were centrifuged at 500 g for 30 min with no brake at room temperature. PBMCs layer was collected and the cells were washed with PBS. For granulocytes (GCs) isolation, double ficoll gradient was used as follows. In a 15-ml falcon tube, 3ml of Histopaque-1119 (Sigma #11,191) was added and then slowly another 3ml Histopaque-1077 (Sigma #10,771) on top of previous to create gradient. Blood was slowly added, diluted 1:1 with PBS and centrifuged as previously mentioned. Top interphase and second interphase were collected in new tubes respectively and washed 1 × with PBS twice.

For in vitro experiments, PBMCs and GCs were isolated from the Case, SLE patients (*n* = 3) and healthy individuals (*n* = 3). SLE patients had active disease. Cells were counted and cultured in RPMI-1640 (GlutaMax, (catalog no. 61870036, Gibco), 10% heat-inactivated FBS catalog no. 10270106, Gibco, 1 × Pen-Strep 100 U/ml and 100 μg/ml, respectively; catalog no. 15140m, Gibco) in a 24-well plate with a density of 1 × 106/ml either under control conditions or with the addition of Etoposide (50mM) for 16h. Cells were then harvested and stained with a) Lympho-panel: CD19-BV510 (#302,241, HIB19), HLA-DR-FITC (#327,006, LN3), CD4-PerCP Cy5.5 (#317,428, OKT4), CD3-PE (#300,308, ΗΙΤ3Α), γΗ2ΑΧ-PE Cy7 (#613,419, 2F3), p-IRF3-APC, CD8-APC Cy7(#344,714, SK1); and b) Myelo-panel: CD16-BV421 (#302,037, 3G8), CD1c-BV510 (#331,533, L161), HLA-DR-FITC (#327,006, LN3), CD15-PerCP Cy5.5 (#323,018, W6D3), CD14-PE (#21,620,144, 18D11, γΗ2ΑΧ-PE Cy7 (#613,419, 2F3),p-IRF3-APC in dilution 1/100 and according to manufacturer’s guide.

### CyToF (Cytometry by Time of Flight)

The Maxpar Human Immune Monitoring Panel Kit (HIM panel, Standard Biotools (SB) Inc., formerly Fluidigm, San Francisco, USA) was used for mass cytometry analysis. This kit contains 29 metal-tagged antibodies against human immune cell surface antigens (Supplementary Table 1). Cell preparation for mass cytometry analysis was performed according to manufacturer instructions and previous studies [31]. Briefly, PBMCs were isolated as previously mentioned and subsequently cryopreserved in freezing medium (FBS/DMSO 10%). For cell staining, PBMCs were thawed in prewarmed RPMI with 10% FBS (medium), washed twice with medium and resuspended in fresh medium. Cells were stained with 1 μM Cisplatin Cell-ID™ (SB) for live/dead cell discrimination and washed with Maxpar cell staining buffer (CSB, from SB), followed by blocking (Human TruStain FcX, from Biolegend). Next, cells were stained for cell surface markers with the HIM panel according to the manufacturer instructions followed by two washes with CSB and fixation (with freshly prepared 1.6% methanol-free formaldehyde solution, from Thermo Scientific) for 20 min at room temperature. Finally, cells were stained in DNA intercalator solution (1:1000 dilution of 125 μM Cell-ID™ Intercalator-Ir, from SB), in Maxpar Fix and Perm buffer (SB) overnight. Next day, cells were washed with CSB buffer and Cell Acquisition Solution (CAS, from SB). Prior to cell acquisition, cells were resuspended with EQ Passport beads (1:10 dilution, from SB). To maximize data quality, the acquisition rate on the Helios™ system (SB) was maintained at a rate of 300 events/s. Normalization of acquired data was performed using Passport beads method with CyTOF software (version 10.7.1014). Data cleanup and sample quality check were performed prior to analysis, using bivariate dot plots with FlowJo™ v10.8 Software (BD Life Sciences, Franklin Lakes, NJ, USA). Then we exported normalized, cleaned fcs files, and used them for downstream analysis (clustering, FlowSOM and dimensionality reduction, tSNE) in R programming environment (version 4.1.0) employing validated, previously published open-source workflows [32]. For additional data analysis, we employed Maxpar Pathsetter™, an automated software specifically designed for streamline analysis of CyTOF data. Pathsetter analysis is a two-step process where initially all events indicative of debris, aggregates, dead cells, and normalization beads are removed and then a comprehensive report that identifies 37 immune cell populations in peripheral blood, utilizing pre-defined gating strategies and definitions is generated [33, 34].

### Immunofluorescence and confocal microscopy

PBMCs were seeded in coverslips pretreated with poly-L lysine, fixed with 4% PFA for 20 min at room temperature (RT) and washed with PBS. Cells were blocked and permeabilized with 1% BSA dissolved in PBS containing 0.1% Triton X-100 (blocking buffer) for 30 min at RT. Next, cell-seeded slides were incubated with primary antibodies and DAPI in blocking buffer at RT for 1h, followed by three washes with PBS containing 0.1% Triton X-100 and then by secondary antibodies for 45 min at RT in dark. Finally, cells were mounted with ProLong™ Diamond Antifade Mountant (Cat. P36961, Thermo Fisher Scientific) and visualized using inverted confocal imaging system Leica SP5. Foci per cell were calculated using a macro developed in Fiji software [35]. The primary antibodies in the immunofluorescence were against γΗ2ΑΧ (Ser139) (1/200), p-ATR (Thr1989) (1/100), p-ATM (Ser1981) (1/100), p-p53 (S15) (1/100) and cleaved Caspase 3 (Asp175) (1/800), and the secondary were Alexa fluor 555 anti-mouse IgG (1/500) and Alexa fluor 488 anti-rabbit IgG (1/500) (Invitrogen). For the analyses, more than 60 cells per human subject (corresponding to a minimum of 4 different fields of the coverslip) were observed for each marker.

### Statistical analysis

Statistical significance was determined by Student’s t-test. Values of *p*-value < 0.05 were considered statistically significant. Data are represented as means ± SEM. Prism 8 software was utilized for statistical analysis.

## Results

### Clinical phenotype and clinical course of patient

**Fig. 1:**
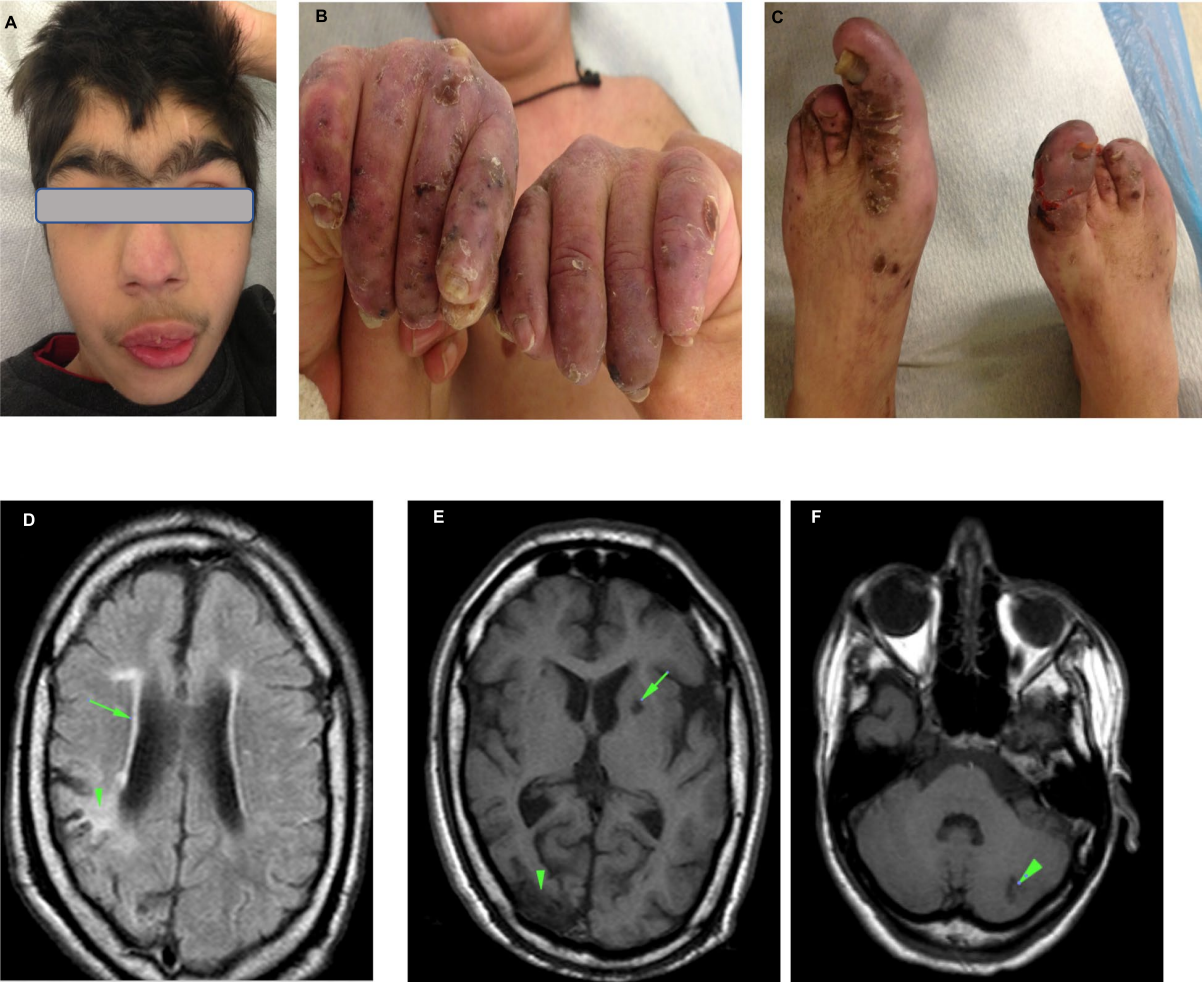
Clinical manifestations of the Case. **A** Synophrys, strabismus, violaceous lesions on the nose; **B-C**. violaceous, scaling lesions of the fingers and toes, resorption of distal phalanges, dystrophic nail changes; **D**. Axial Flair sequence showing reduction of the cerebral white matter with high signal suggestive of diffuse inhomogeneous leukoencephalopathy (arrow). In the cerebral parietal cortex a wedge-shaped microinfarction with gliosis (arrowhead); **E–F**. Axial T1 weighted sequence showing multiple low signal (arrows) abnormalities in cerebral and cerebellum cortex that represent infarctions related to the widespread microangiopathy

A 20-year old male of Greek origin presented since early infancy with violaceous, scaling lesions of the fingers and toes, resorption of distal phalanges, dystrophic nail changes and, violaceous lesions on the nose that worsened during the cold season. He had a history of chilblain lesions of fingers and toes since infancy. In addition, he exhibited a short stature (growth below the 3rd percentile), severe psychomotor retardation, microcephaly, hypertrichosis, synophrys, arched palate, strabismus, spasticity, scoliosis, hearing loss, cryptorchidism and unilateral vesicoureteral reflux with kidney scarring, leading to the clinical diagnosis of Cornelia de Lange syndrome (CdLS) at the age of 7 years (Fig. 1A-C). Laboratory investigations revealed intermittent mild leucopenia and thrombocytopenia and positive anti-thyroglobulin antibodies. Anti-nuclear antibodies were negative, and C3—C4 complement serum levels were in normal range. A cerebral MRI showed infarctions in the cerebral and cerebellum cortex, microinfarction with gliosis in the cerebral parietal cortex and diffuse inhomogeneous leukoencephalopathy (Fig. 1D-F). At the age of 21 he experienced an episode of bloody stools. A colonoscopy showed mucosal lesions compatibles with ischemic colitis (mucosal erythema, oedema, ulcerations, submucosal hemorrhage). He received hydroxychloroquine, low dose corticosteroids (2.5-5mg/day), aspirin, thiocolchicoside, amlodipine and lansoprazole with mild improvement of the cutaneous involvement. Additionally, he received nebivolol following an episode of sinus tachycardia, and baclofen to alleviate spasticity. The patient's family history was not known as the patient was adopted and the biological family records were not available. Baricitinib (JAK1/2 inhibitor) was administered as initial disease-specific therapy (accompanied by corticosteroids in tapering and hydroxychloroquine) after initial visits of the case (baseline clinical evaluation and sampling took place prior to JAKi addition) and the patient presented adequate response. Due to the resemblance of clinical phenotype between AGS and SLE (lupus is considered an interferonopathy), we utilized as control disease a cohort of lupus patients (*n* = 8), exhibiting active disease, ANA positivity (7/8), anti-dsDNA positivity (6/8) and low complement (7/8). Two patients were naïve of therapy at the time of sampling while the rest were treated with hydroxychloroquine, glucocorticosteroids and immunosuppressive agents (but not biologicals). SLE patients presented with various disease manifestations, among them arthritis and serositis and renal, neurological and hematological involvement.

### Whole exome-sequencing establishes two distinct genetic variants

**Fig. 2:**
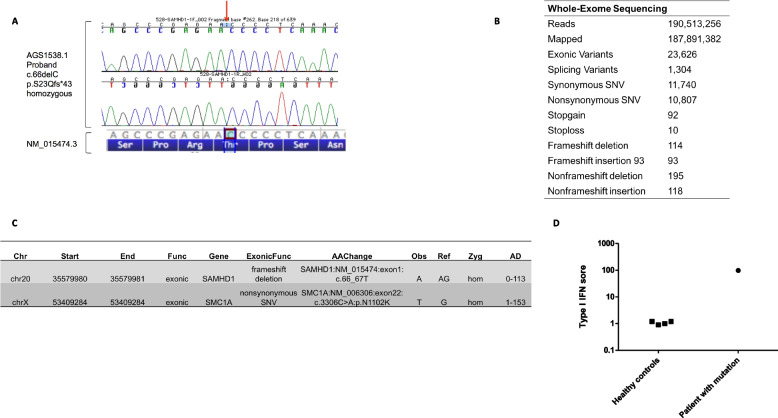
Genetic analysis of the Case and interferon score. **A** Sanger sequencing confirming c.66delC mutation in SAMHD1 in the proband **B**. Whole-Exome Sequencing was performed in a patient with CdL/AGS syndrome. **C**. CdL causal mutation is defined as a novel one with unknown functionality until now (*MX1*AGS causing mutation is also characterized as novel (*SAMHD1,* NM_015474.3:c.66del:p.(Ser23Glnfs*43), homozygous) **D**. IFN score (MX-1, IFIT-1, IFI44) in our patient and healthy individuals

Considering the clinical phenotype suggestive of both Aicardi-Goutières syndrome (AGS) and Cornelia de Lange syndrome (CdL), we proceeded to depict the genetic map of the patient. A targeted next-generation sequencing panel for AGS genes revealed a novel homozygous variant (frameshift deletion) c.66delC; p.Ser23Glnfs*43; NM_015474.3 in *SAMHD1* gene (Fig. 2A). The variant was confirmed by Sanger sequencing. This variant creates a premature translational stop signal. It is expected to result in an absent or disrupted protein product. The variant is not present in Genome Aggregation Database (gnomAD). This variant is listed in ClinVar as pathogenic (Variation ID: 858,736) and is characterized as pathogenic by the ACMG classification (PMID 31534211).

To confirm this finding and further investigate the genetic background, whole exome sequencing (WES) was performed. WES confirmed the variant in SAMHD1 gene and identified a novel hemizygous missense variant in SMC1A gene (NM_006306.3:c.3306C > A p.(Asn1102Lys)). This variant is listed in ClinVar as variant of unknown significance VUS (PMID 31534211) (Fig. 2B, C).

Together these findings confirm that our patient bears genetic background leading to the presentation of two syndromes simultaneously, one interferonopathy (AGS) and one cohesinopathy (CdL).

### Genetic variants of the patient alter transcriptional output towards an autoimmune profile

Aicardi-Goutières syndrome is an interferonopathy characterized by marked enhancement of type I interferon (IFN) genes expression. Thus, we next evaluated IFN score by measuring gene expression of 3 interferon stimulated genes (ISGs), namely MX-1, IFIT-1 and IFI44, in the peripheral blood of the patient compared to age/sex matched healthy individuals (*n* = 4). IFN score was found to be almost 98-fold higher than the mean score of healthy controls, mirroring the deregulation of the respective pathway (Fig. 2D).

**Fig. 3:**
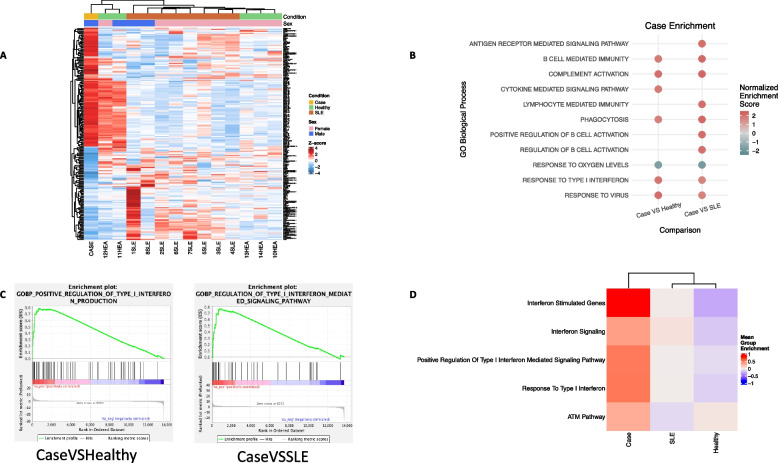
Transcriptome profiling of peripheral monocytes of the Case reflects aberrations of both interferonopathy and cohesinopathy. **A**.Heatmap of the expression z-score of the top 200 DE genes (as ranked by absolute logFC) between Case vs. SLE patients. **B** Bubble plot of GSEA analysis representing enriched pathways associated with the Hallmark database in comparisons of DEGs Case/healthy and Case/SLE. The size of the bubbles represents the statistical significance. **C** GSEA plots for key pathways involved in the pathogenic features in comparisons of DEGs Case/healthy and Case/SLE. **D** Heatmap depicting relative enrichment of key pathogenic enriched pathways in Case, SLE patients and healthy controls

As monocytes are key effector cells in interferonopathies and in SLE [36, 37], we further studied the transcriptional aberrations of CD14^+^ monocytes by performing mRNA-sequencing. This expression profile was compared to that of healthy controls (*n* = 5) and SLE patients (*n* = 8) (Supplementary Table 2). Transcriptome profiling demonstrated 424 differentially expressed genes (DEGs) to be upregulated in the Case compared to healthy controls, while 268 were downregulated. On the other hand, 435 DEGs were upregulated in the Case compared to SLE patients, while 264 genes were downregulated **(**Fig. 3A**, **Supplementary Table 3). Overall, there are clear transcriptional differences between the Case and the SLE group, which we further investigated at a pathway level.

Gene-set enrichment analysis (GSEA) on DEGs of Case compared to healthy controls and various immune pathways showed complement activation and type I IFN signaling. The same analysis on Case compared to SLE patients, showed that antigen receptor-mediated signaling, B-cell activation and type I IFN signaling stand out **(**Fig. 3B**).** Both comparisons highlight type I IFN pathway as one of the main transcriptionally reprogrammed pathways **(**Fig. 3C, Supplementary Fig. 1). Finally, focusing further on aberrant pathways in the Case compared to healthy and autoimmune state, we used gene-set variation analysis (GSVA) for ISGs and DNA-damage repair (DDR) genes. Type I IFN pathway is utmost activated at Case (higher than both SLE patients and healthy individuals), while selected members of DDR pathway (ATM pathway) show aberrant expression compared to healthy controls **(**Fig. 3D**, **Supplementary Fig. 2A). Distinct transcriptional pathways are shared between CasevsHealthy and CasevsSLE comparisons, mainly including type I IFN pathway, virus response, pyroptosis and adaptive immunity. Pathways playing role i) exclusively to CasevsHealhty comparison include receptor signaling, chemotaxis, phagocytosis while ii) exclusively to CasevsSLE comparison contain B cell mediated immunity, immunoglobulin mediated immunity and complement activation (Supplementary Fig. 2B). Together, these data suggest that the transcriptome of peripheral monocytes of the Case is marked by aberrant expression of type I IFN signaling and DNA-damage response, both pathways being central to the inflammatory and autoimmune phenotype of the patient [36, 38].

### Immune phenotyping reveals expanded granulocytic populations and dampened T effector responses

**Fig. 4:**
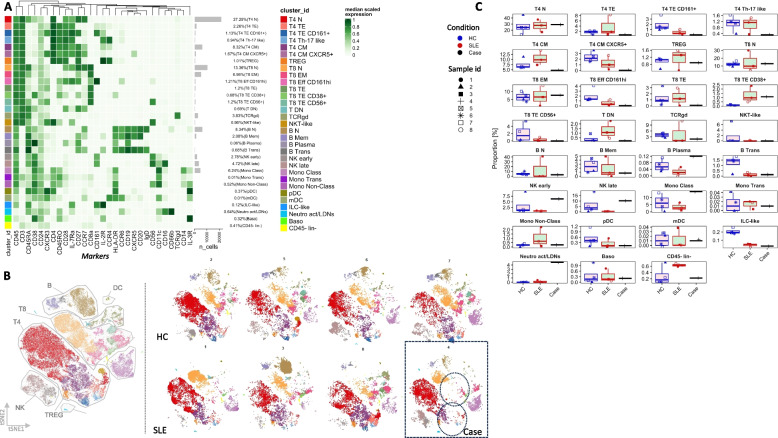
Immunophenotyping analysis of PBMCs from the Case, SLE patients and healthy controls expanded granulocytic populations and dampened T effector populations. **A** Heatmap of the median marker intensities (Arcsinh, 0–1 transformed marker median expression calculated over cellular events from all the samples in the data set, Healthy Controls, HC, *n* = 4, SLE patients, *n* = 3 and the Case) of the 29 markers across the 31 cell populations obtained with FlowSOM (cluster id, colour annotated). The intensity of the green colour is indicative of low to high marker expression respectively. Hierarchical similarity between the expression of the 29 markers is noted with a dendrogram on the top (Euclidean distance; average linkage). **B** Dimensionality reduction analysis, t-SNE plot, visualizing cluster assignments of cell subtypes, representative of all samples (left panel) and each sample individually (right panel). **C** Boxplots showing relative frequency (proportion, of live singlet PBMCs) of the 31 identified FlowSOM metaclusters for the three conditions, HC, SLE and the Case

Next, we used mass cytometry (Cytometry by time-of-flight, CyTOF) to gain high resolution immune phenotyping of the PBMC landscape of the Case compared to SLE patients (*n* = 3) and healthy controls (*n* = 4). To this end, we used a 29-marker multiplex CyTOF panel to immunophenotype a broad spectrum of immune cells like T, B and NK lymphocytes, myeloid cells as well as their subpopulations (Fig. 4). Initially, we performed clustering with FlowSOM algorithm to identify and annotate 31 distinct cellular identities in our dataset (clusters, Fig. 4A) based on combinations of the expression of extracellular proteins-markers on PBMCs such as classical lineage markers CD3, CD4, CD8, CD19, CD20, CD11c, CD56, CD14, CD16 and CD66b as well as markers indicative of activation and homing (Supplementary Table 4). Then, we used the dimensionality reduction algorithm tSNE to visualize the resulting clusters on a 2D map, on which annotated clusters are also colored to aid exploration of the data (Fig. 4B). Using a reference map that includes all the events from all the samples that were processed by the algorithm (Fig. 4B, *left panel*), we explored the tSNE maps from all the samples in our dataset (HC *n* = 4, SLE *n* = 3, and the Case, Fig. 4B, *right panel*). This exploratory analysis revealed that the Case demonstrates mixed immune features, either resembling SLE or HCs, but most importantly some unique characteristics that are not mirrored in either group. The most profound and unique phenotype evident from these maps in the PBMC landscape of the Case was the absence of effector/activated subpopulations of CD4 + and CD8 + T lymphocytes (Fig. 4B, *right panel*—*dotted circles*).

These observations were confirmed when we compared in detail the frequencies of all the identified clusters in our dataset (Fig. 4C**, **Boxplots). More specifically, the Case exhibited lower frequencies of clusters representing activated CD4^+^ T lymphocytes (T4) like *T4 TE* (CD45RA^−^ CD45RO^+^), *T4 Th-17 like* (CXCR3^−^ CCR6^+^ CXCR5^−^ CCR4^+^), *T4 CM* (CCR7^+^ CD45RA^−^ CD45RO^+^) as well as lower frequencies of clusters representing activated CD8^+^ T lymphocytes (T8), like *T8 TE* (CCR7^−^CD45RA^−^CD45RO^+^) and *T8 Eff CD161*^*hi*^ (high expression of killer cell lectin-like receptor subfamily B, member 1, KLRB1, CD161) compared to SLE patients and HCs. Moreover, the Case showed higher frequencies of NK subsets (*NK early,* CD56^+^ CD16^−^ and *NK late,* CD56^+^ CD16^+^), B cell plasmablasts (B plasma CD38^++^ CD27^+^ IgD^−^ CD20^−^ CXCR5^−^) and Classical Monocytes (Class Mono, CD16^−^ CD14^++^). Finally, the unique features of the Case peripheral immune profile also included higher frequencies of cells of the granulocytic lineage that express CD66b and CD16. These cells are most likely activated Neutrophils or Low-Density Granulocytes (LDGs) that due to their morphological changes during activation they may be co-purified with mononuclear cells during Ficoll separation.

In further analysis, the Case exhibited similarities to the SLE patients PBMC immune profile. The similarities were the lower frequencies of *T4 TE CD161*^+^ and *T4 CM CXCR5*^+^ as well as lower frequencies of *T8 TE CD56* + *,* compared to HCs. Interestingly, the only identified cluster of activated of T lymphocytes that was presented in higher abundance both in the Case and SLE patients compared to HCs was the *T8 TE CD38*^+^ cluster. In addition, the Case presented lower frequencies of the *B trans* cluster (B transitional cells, CD38^++^ CD27^+^IgD^+^ CD20^+^ CXCR5^+^ CD45RA^+^ CD25^+^) and lower frequencies of *pDC* cluster (plasmacytoid Dendritic Cells, CD11c^−^ CD123^+^) and ILC-like cells (Innate Lymphoid Cells, CD45^low^ CD3^−^ CD19^−^ CD25^high^CD127^+^ CD161^+^ CD45RA^+^ CD11c^−^), mirroring SLE patients compared to HCs. This analysis also revealed no differences in circulating T regulatory cells, TREG (CD25^+^CCR4^+^ CD127^−^) between the Case, SLE patients and HCs.

Finally, since our analysis was based on manual annotation of the resulting clusters, we sought to validate these results using Pathsetter which is an automated workflow for the analysis of high dimensional CyTOF data [33, 34]. The automated Pathsetter analysis validated our findings as we were able to observe similar patterns of differences of immune cell frequencies among HCs, SLE patients and the Case. Pathsetter analysis also revealed that within the Th cell compartment the Case presented very low (almost absent) frequencies of Th1-like (CXCR3^+^ CCR6^−^ CXCR5^−^) and Th17-like cells (CXCR3^−^ CCR6^+^ CXCR5^−^ CCR4^+^) compared to the other groups, whereas Th2-like cells (CXCR3^−^ CCR6^−^ CXCR5^−^ CCR4^+^) appear in similar frequencies across the three groups (Supplementary Fig. 3).

### The autoimmune phenotype of the case is characterized by increased apoptosis through DNA-damage response

**Fig. 5:**
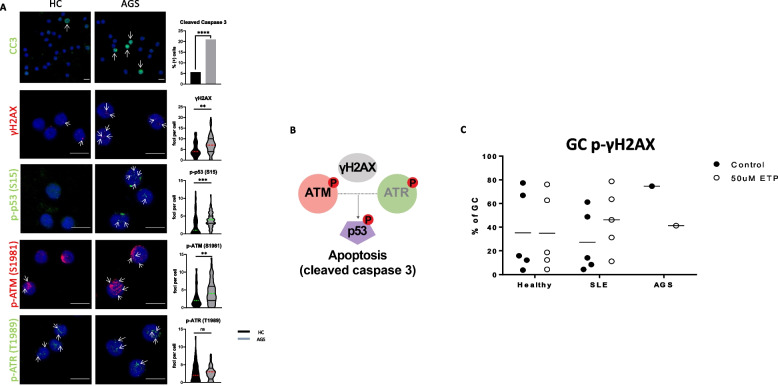
Increased apoptosis in AGS correlates with ATM-mediated DNA damage response A. Apoptosis levels as determined by assessment of cleaved caspase 3 (CC3) and DNA damage response via examination of the following phosphorylated proteins in PBMCs under immunofluorescence confocal microscopy: γΗ2ΑΧ, p53 (S15), ATM (S1981) and ATR (T1989) in AGS and HC (*n* = 1/condition). Analyzed results for γΗ2ΑΧ, p-p53, p-ATM and p-ATR are depicted as foci per cell. For CC3 staining, positive signal is depicted with green and cells are quantified as positive or negative utilizing dapi nuclear staining (blue). Statistical significance was obtained by unpaired Student’s t test, *p* ≥ 0.05 (ns), *p < 0.05, **p < 0.01 and ***p < 0.001. Scale bar: 10 μM. (B) **B** Schematic representation of the results indicating that apoptosis occurs due to DNA damage response. **C** Granulocytes (derived of peripheral blood) from CdL/AGS patient although exhibit higher basal level of DNA damage compared to healthy individuals and SLE patients, p-γΗ2ΑΧ levels are approximately equal to the controls, after induction of DNA breaks with 50 uM of Etoposide

DNA-damage response is a key characteristic inflammation driven event in autoimmune milieu. Specifically, various studies have demonstrated that aberrant DDR is present in various disease setups, such as SLE, RA and AGS [38–43]. To further explore this, we performed an extended analysis of DDR-related apoptosis pathway on PBMCs of the Case and healthy control, monitoring major signal transducers such as phospho-ATR, phosphor-ATM, phospho-p-53, γ-H2AX and cleaved caspase 3 through immunofluorescence confocal microscopy **(**Fig. 5A-B**).** Our data demonstrate that DDR pathway is activated in Case compared to healthy control, as higher levels of p-ATM, p-p53 and γ-H2AX were determined. To evaluate apoptosis in the Case, we assessed cleaved caspase 3 levels and found them significantly higher compared to steady state.

In order to interrogate the capacity of various cell subtypes to respond to DNA damage, we next used an in vitro system where cells were cultured with or without the presence of 50 μM etoposide (a strong inducer of DNA-breaks) and then levels of γH2AX were measured by flow cytometry. CD15^+^CD14^+^ granulocytes of AGS patient presented with higher levels of γH2AX from both healthy individuals and SLE counterparts, reflecting a significantly higher basal level DNA damage. In spite of this, after etoposide incubation only SLE granulocytes exhibit induced levels of γ-H2AX, while DNA-damage levels fall to levels equal to healthy control in AGS patient **(**Fig. 5C**).** This result might be explained by increased apoptosis levels of PBMCs in AGS Case, as described previously. Other key cell subsets, such as B-cells, T-cells, dendritic cells and distinct monocytic subsets did not show the same trend (Supplementary Fig. 4). Together, our data corroborate that increased apoptosis, driven by enhanced DNA-damage response, takes place in the peripheral blood of AGS patient and is mediated via the ATM pathway, likely fueling the inflammatory phenotype of the disease.

## Discussion

Herein, we describe a Case with an extreme genetic phenotype of two simultaneous genetic lesions, a Type I interferonopathy (Aicardi-Goutiéres Syndrome, AGS) combined with Cornelia de Lange Syndrome (CdLS)—a cohesinopathy. The combination of the two genetic lesions leads to broad alterations in immunophenotype and transcriptome. DNA damage response and type I interferon signaling are the key pathways driving the disease manifestations in our Case. Accordingly, we will outline mechanistic data known in the field and use them to suggest how mutations present in our Case may lead to alterations in DNA damage, IFN signaling and immune regulation.

Our Case has the unique feature to combine transcriptional programs, affected by both disorganization of chromatin environment through cohesin complex defects and type I IFN induction. The genetic aberrancies bared by this Case, modulate both characteristics of immune cell types of myeloid as well as lymphoid lineage. Cohesionopathies and interferonopathies find common ground to the induction and deficiency of DNA damage response, as shown by our results too, combined with genome instability. AGS syndrome is characterized by continuous stimulation of autoreactivity through interferon gene expression. On the other hand, the role of cohesin deficiency through diversification of chromatin compartmentalization, on top of an already autoinflammatory/autoimmune skewed gene expression program, might be even more profoundly affected with the potential of being detrimental. Specific gene modules, differentially expressed in AGS, are expected to shift localization due to the deregulated boundaries of chromatin topology, interfering combinatorically with their expression.

While interferonopathies are well studied in SLE context, the role of SMC1A mutations and the cohesinopathies in immune system is not fully known. Members of cohesin family are implicated in chromatid segregation and gene regulation, thus we predict that constitutive gene transcription programs of immune cells would be perturbed in these patients. Our transcriptomic analysis has pinpointed various immune-associated aberrations, such as B-cell specific immune response, complement activation, T-cell related cytokine signaling, antigen-receptor mediated signaling and IFN signaling. Moreover, mass cytometry analysis revealed the absence of effector/activated CD4 + and CD8 + T cells. These results are corroborated by recent literature, depicting the vast impact of cohesin function to immune system. Specifically, cohesin mediates TLR-related molecular cascades in cells of myeloid lineage, dictates IFNG expression in CD4 + T-cells and regulates expression of Th2 cytokines IL-4, IL-5, and IL-13 [44–47]. Moreover, SMC1A expression plays a crucial role in function of various T cell populations while it may be a predictive marker of immune checkpoint inhibitor therapy, in a tumor setup [48]. The mechanism which SMC1A seems to utilize for immune-specific gene expression is the differential localization of the CTCF/cohesin complex, demarcating active from silent chromatin. Through this mechanism, deregulation of cohesin complexes dramatically impairs monocyte differentiation in humans [49]. Additionally, deficiency in cohesin complex members results to DNA damage and induces interferon expression via the cGAS-STING pathway [50]. Lately, cohesin complex has been shown also to regulate enhancer mediated inducible expression in innate immune genes [51]. Together, these data suggest that the SMC1A mutation identified in our Case may be contributing and amplifying the immune aberrancies.

The immune landscape of patients with interferonopathies is fundamentally compromised by the set of genes induced by type I IFN. We have documented by IFN-score and transcriptomic analysis the perturbed IFN signaling in the Case, compared to healthy individuals and SLE patients. Furthermore, RNA-sequencing analysis and in vitro DDR experiments have linked this deregulated IFN cascade with atypical DNA damage signaling. Interferonopathies are characterized by both these molecular patterns. Primarily, loss-of-function of TREX1 leads to activation of type I interferon signaling mediated through the cGAS/STING pathway [52]. Fluctuations in expression of the same gene causes cellular senescence and perturbs DDR mechanism [53]. Our Case exhibits a mutation on SAMHD1 gene. SAMHD1 maintains genome integrity through securing proper DNA synthesis, while its absence induces IFN activation and genome instability [43]. On top of the previous, SAMHD1 is implicated in DNA damage levels, while repair of double strand breaks via homologous recombination is one of its functions [54, 55]. Key immune cell types are influenced by SAMHD1 deficiency. Specifically, SAMHD1-deficient monocytes autonomously initiate type I IFN and induction of ISGs [56]. Downregulation or loss of SAMHD1 expression augments the susceptibility of myeloid-derived cells or resting CD4 + cells to infection [57, 58]. SAMHD1 knockdown drives innate immune activation through increased expression of RNA-sensors and ISGs [59].

Mass cytometry (CyTOF) has transformed our understanding of the immune system, offering unparalleled detail and insight into its complex dynamics, particularly in rare clinical cases where conventional diagnostics may not fully reveal the underlying immune dysregulation. This advanced immune profiling is essential for uncovering disease mechanisms, informing targeted treatment strategies, and enhancing patient care. Our data, derived from comprehensive immune profiling using CyTOF, have enabled us to formulate two distinct hypotheses regarding the underlying immune mechanisms at play in this rare clinical case. The Case demonstrated a unique immune profile, mainly characterized by the absence of effector/activated subpopulations of CD4 + and CD8 + T lymphocytes, which are crucial for an effective immune response.

Our alternative hypothesis is that these findings may indicate an impaired adaptive immune response in our Case. This could suggest a unique type of autoimmune or immune dysregulation syndrome, hindering the patient's ability to effectively combat infections or other immune challenges, thereby aggravating symptoms. Moreover, the Case exhibits elevated levels of NK cell subsets, B cell plasmablasts, classical monocytes, and notably, activated, or low-density granulocytes, suggesting a unique pathological shift in the immune landscape. While sharing some immune characteristics with SLE patients, such as reduced frequencies of specific T cell subsets and comparable B cell and dendritic cell profiles, the Case’s distinct immune cell profile, especially the lack of activated T cell populations, underscores a unique immune dysregulation potentially driving this disease that has led to immune exhaustion/senescence and an overall decrease in immune activation capacity, or an underlying immune regulatory disorder*.*

The observed immune phenotype could also relate to homing of these cells to specific tissues in response to infection, inflammation, or autoimmune activity. More specifically, lymphocyte homing involves chemokines, adhesion molecules, and receptors like CCR7 and CXCR3, directing T cells to damaged or infected tissues [60, 61]. Targeted lymphocyte migration is essential for pathogen clearance and tissue repair [62, 63]. In autoimmune diseases such as SLE, abnormal lymphocyte homing, driven by chemokine receptor dysregulation, directs autoreactive T cells to organs, causing tissue damage and inflammation [64, 65]. Given the unique immune profile of the Case, with a specific absence of activated CD4^+^ and CD8^+^ T lymphocyte subpopulations in the peripheral blood, it is plausible to consider that these cells may be homing to specific tissues in response to signals of infection or damage. This could explain their reduced presence in the peripheral circulation. The higher frequencies of other immune cells, such as NK cells, B cell plasmablasts, and activated neutrophils, further suggest an active immune response that may be localized to specific tissues rather than being systemic. The possibility that these lymphocytes have homed to tissues of infection or damage in the Case presents an important avenue for investigation. Imaging studies or tissue biopsies, combined with immunohistochemistry or in situ hybridization, could provide direct evidence of lymphocyte infiltration into specific tissues. Understanding the dynamics of lymphocyte trafficking in this context could provide valuable insights into the underlying pathology of the Case and potentially guide therapeutic strategies aimed at modulating immune cell migration and function.

We also observed that increased apoptosis and ATM-mediated DNA damage response take place in the periphery of this Case. We have already commented on the relationship of DDR with both AGS and CdLS. Loss of ATM signaling in an AGS background seems to deteriorate neurodegeneration documented in these patients, through genomic instability [66]. Moreover, cells derived from AGS patients exhibit chronic ATM-dependent checkpoint activation of cell cycle, connecting this finding to autoimmunity and inflammation [67]. Deficiency of various components of the cohesin complex leads to enhanced apoptosis through dysregulation of cell cycle genes, an event connected to aberrant neural development of these patients [68]. In interferonopathies, constant nucleic acid sensing drives both T regulatory cells and B cell into apoptosis, the latter ones due to impaired maturation [69, 70]. SAMHD1 is a regulator of STING-mediated apoptosis in human monocytes, in viral infection [71]. The strong stimulus towards activation of ATM pathway, DDR activation and increased apoptosis is profoundly augmented by both genetic variants of our Case.

Limitations of this study include: 1) the fact that is based on a single case of AGS patients, which has an important impact on the statistical analysis as well as the generalization of our results. No disease controls of patients presenting with only one of two syndromes were included, as these cases are rare and literature is limited. 2) Whole exome sequencing is utilized for the description of the genetic component, while a detailed analysis of whole genome is missing. 3) The lack of single-cell RNA sequencing analysis, although this study contains a broad immune phenotyping in single cell level. 4) No post JAKi-treatment IFN or DNA damage related transcriptomic studies were performed on the Case, but it would be of great interest to document a reversal of one or both molecular phenotypes after therapy. Not withstanding these limitations, the detailed analysis of this case may provide useful information for further analyses.

## Conclusion

Two distinct at first unrelated genetic lesions but apparently synergizing in causing genomic damage and interferon response accompanied by broad immune phenotyping with granulocytic skewing and absence of activated T cells compatible with chronic antigenic stimulation and homing at sites of inflammation. Further studies are needed to better define the role of cohesins in autoimmunity and inflammation.

## Supplementary Information

Supplementary Material 1: Table 1. The list of antibodies (all anti-human) of the 29-plex CyTOF analysis.

Supplementary Material 2: Table 2. Clinical parameters of the patients participated in the RNA-seq experiment.

Supplementary Material 3: Table 3. List of Differential Expressed Genes (RNA-seq) on comparisons CasevsHealthy and CasevsSLE (*P*-value <0.05 and |FC|³1.5).

Supplementary Material 4: Table 4. CyToF Analysis Pathsetter Reports.

Supplementary Material 5: Supplementary Figure 1. A. Cumulative IFN-score (Reactome - IFN signaling) for Case, SLE patients and healthy controls. B. Gene-specific expression of genes utilized for IFN-score in Case, SLE patients and healthy controls. C. Heatmap of the expression of IFN-related genes in Case, SLE patients and healthy controls.

Supplementary Material 6: Supplementary Figure 2. A. Heatmap showing the enrichment in WikiPathways DNA Damage Response, GOBP: Interferon Mediated Signaling Pathway and the publication-derived Interferon Stimulated Genes signature, as calculated by GSVA. B. Venn diagram of differentially expressed genes between 1) CasevsHealthy and 2) CasevsSLE.

Supplementary Material 7: Supplementrary Figure 3. Automated Immunophenotyping analysis of PBMCs from the Case, SLE patients and healthy controls with the Maxpar Pathsetter workflow. Boxplots showing relative frequency (% positive cells of live singlet PBMCs) of the identified cell types from the automated workflow for the three conditions, HC, SLE and the Case (CM, Central Memory, EM, Effector Memory, TE, Terminal Effector, TREG, T regulatory).

Supplementary Material 8: Supplementary Figure 4. Percentage of different immune cell types (B cells, T cells, Dendritic Cells, classical monocytes, non-classical monocytes, intermediate monocytes) with high abundance of phospho-γH2AX and/or phospho-IRF3 following 16h of culture +/- 50μM Etoposide (ETP).

## Data Availability

Data are available on reasonable request. RNA sequencing (RNA-seq) and Genome Exome Sequencing data have been deposited to the EGA (European Genome-Phenome Archive) database under study number EGAS00001007829.

## Abbreviations

ACMG: American College of Medical Genetics
AGS: Aicardi-Goutières Syndrome
ANNOVAR: ANNOtate VARiation
ATM: Ataxia-telangiectasia mutated
BSA: Bovine Serum Albumin
BWA: Burrows–Wheeler Aligner
CAS: Cell Acquisition Buffer
CdLS: Cornelia de Lange Syndrome
cGAS/STING: cyclic GMP-AMP synthase (cGAS)-stimulator of interferon genes (STING)
CSB: Cell Staining Buffer
CyToF: Cytometry by time of flight
DAPI: 4′,6-diamidino-2-phenylindole
DDR: DNA Damage Response
DEGs: Differentially Expressed Genes
DMSO: Dimethyl sulfoxide
FBS: Fetal Bovine Serum
GATK: Genome Analysis Tool Kit
GCs: Granulocytes
gnomAD: Genome Aggregation Database
GSEA: Gene Set Enrichment Analysis
GSVA: Gene Set Variation Analysis
HCs: Healthy Controls
IEIs: Inborn Errors of Immunity
IFN: Interferon
ISGs: Interferon Stimulated Genes
JAKs: Janus Kinase Inhibitors
LDGs: Low Density Granulocytes
MRI: Magnetic Resonance Imaging
MSigDB: Molecular Signatures Database
PBMCs: Peripheral Blood Mononuclear Cells
PBS: Phosphate Buffered Saline
qRT-PCR: quantitative Real Time – Polymerase Chain Reaction
RT: Room Temperature
SLE: Systemic Lupus Erythematosus
tSNE: t-distributed Stochastic Neighbor Embedding
WES: Whole Exome Sequencing
WGS: Whole Genome Sequencing
