# Supplementary material for: Increased interferon I signaling, DNA damage response and evidence of T-cell exhaustion in a patient with combined interferonopathy (Aicardi-Goutières Syndrome, AGS) and cohesinopathy (Cornelia de Lange Syndrome, CdLS)
Source: Pediatr Rheumatol Online J. 2025 Jan 27;23:11. doi: 10.1186/s12969-024-01050-7 (PMC11770959; doi:10.1186/s12969-024-01050-7)
Supplement: Supplementary file 1 — Supplementary Material 1: Table 1. The list of antibodies (all anti-human) of the 29-plex CyTOF analysis. [file 12969_2024_1050_MOESM1_ESM.docx]

| Antibody  (*anti-human*) | Clone | Mass | Amount used/sample (ul) | Dilution in staining |
| --- | --- | --- | --- | --- |
| Anti-CD45 | HI30 | 89Y | 1 | 1: 100 |
| Anti-CD19 | HIB19 | 142Nd | 1 | 1: 100 |
| Anti- CD127/IL-7Ra | A019D5 | 143Nd | 1 | 1: 100 |
| Anti-CD38 | HIT2 | 144Nd | 1 | 1: 100 |
| Anti-IgD | IA6-2 | 146Nd | 1 | 1: 100 |
| Anti-CD11c | Bu15 | 147Sm | 1 | 1: 100 |
| Anti-CD16 | 3G8 | 148Nd | 1 | 1: 100 |
| Anti-CD194/CCR4 | L291H4 | 149Sm | 1 | 1: 100 |
| Anti-CD123/IL-3R | 6H6 | 151Eu | 1 | 1: 100 |
| Anti-TCRgd | 11F2 | 152Sm | 1 | 1: 100 |
| Anti-CD185/CXCR5 | RF8B2 | 153Eu | 1 | 1: 100 |
| Anti-CD3 | UCTH1 | 154Sm | 1 | 1: 100 |
| Anti-CD45RA | HI100 | 155Gd | 1 | 1: 100 |
| Anti-CD27 | L128 | 158Gd | 1 | 1: 100 |
| Anti-CD28 | CD28.2 | 160Gd | 1 | 1: 100 |
| Anti-CD66b | 80H3 | 162Dy | 1 | 1: 100 |
| Anti-CD183/CXCR3 | G025H7 | 163Dy | 1 | 1: 100 |
| Anti-CD161 | HP-3G10 | 164Dy | 1 | 1: 100 |
| Anti-CD45RO | UCHL1 | 165Ho | 1 | 1: 100 |
| Anti-CD24 | ML5 | 166Er | 1 | 1: 100 |
| Anti-CD197/CCR7 | G043H7 | 167Er | 1 | 1: 100 |
| Anti-CD8 | SK1 | 168Er | 1 | 1: 100 |
| Anti-CD25 | 2A3 | 169Tm | 1 | 1: 100 |
| Anti-CD20 | 2H7 | 171Yb | 1 | 1: 100 |
| Anti-HLA-DR | L243 | 173Yb | 1 | 1: 100 |
| Anti-CD4 | SK3 | 174Yb | 1 | 1: 100 |
| Anti-CD56 | NCAM16.2 | 176Yb | 1 | 1: 100 |
| Anti-CD196/CCR6 | G034E3 | 141Pr | 2 | 1:50 |
| Anti-CD14 | M5E2 | 175Lu | 2 | 1:50 |

**Supplementary table 1**. The list of antibodies (all anti-human) of the 29-plex CyTOF analysis and are included in the Maxpar Human Immune Monitoring Panel Kit (Standard Biotools), showing the target antigen, antibody clone, metal tag, amount used (in ul) per sample and final dilution in the staining antibody cocktail.
