# Supplementary material for: Increased interferon I signaling, DNA damage response and evidence of T-cell exhaustion in a patient with combined interferonopathy (Aicardi-Goutières Syndrome, AGS) and cohesinopathy (Cornelia de Lange Syndrome, CdLS)
Source: Pediatr Rheumatol Online J. 2025 Jan 27;23:11. doi: 10.1186/s12969-024-01050-7 (PMC11770959; doi:10.1186/s12969-024-01050-7)
Supplement: Supplementary file 4 — Supplementary Material 4: Table 4. CyToF Analysis Pathsetter Reports. [file 12969_2024_1050_MOESM4_ESM.pdf]

FLUIDIGM

Maxpar® Pathsetter™ Report

Powered by GemStone™

FCS File: AB1_1_1_1_cleaned.fcs

Sample 01 - SLE

	Count	*	% Intact live	Parent	% Parent
Intact Live Cells	186336		100	All events	96.98
Lymphocytes	164039		88.03	Intact Live	88.03
CD3 T Cells	159117		85.39	Lymphocytes	97.00
CD8 T Cells	62019		33.28	CD3 T Cells	38.98
Naive	57957		31.10	CD8 T Cells	93.45
Central memory	1031		0.55	CD8 T Cells	1.66
Effector memory	403		0.22	CD8 T Cells	0.65
Terminal effector	2628		1.41	CD8 T Cells	4.24
CD4 T Cells	94553		50.74	CD3 T Cells	59.42
Naive	54832		29.43	CD4 T Cells	57.99
Central memory	10310		5.53	CD4 T Cells	10.90
Effector memory	23163		12.43	CD4 T Cells	24.50
Terminal effector	6248		3.35	CD4 T Cells	6.61
Treg	3875		2.08	CD4 T cells	4.10
Th1-like	989		0.53	CD4 T cells	1.05
Th2-like	11014		5.91	CD4 T cells	11.65
Th17-like	1043		0.56	CD4 T cells	1.10
Gamma Delta T Cells	2330		1.25	CD3 T Cells	1.46
MAIT & NKT CD4- Cells	215		0.12	CD3 T Cells	0.14
B Cells	41	*	0.02	Lymphocytes	0.02
Naive	0	*	0	B Cells	0
Memory	0	*	0	B Cells	0
Plasmablasts	41	*	0.02	B Cells	100
NK Cells	4881		2.62	Lymphocytes	2.98
Early NK	1609		0.86	NK Cells	32.96
Late NK	3272		1.76	NK Cells	67.04
Monocytes	8886		4.77	Intact Live	4.77
Classical	8181		4.39	Monocytes	92.07
Transitional	618		0.33	Monocytes	6.95
Non-classical	87	*	0.05	Monocytes	0.98
Dendritic Cells	4824		2.59	Intact Live	2.59
pDC	1061		0.57	Dendritic Cells	21.99
mDC	3763		2.02	Dendritic Cells	78.01
Granulocytes	2953		1.58	Intact Live	1.58
Neutrophils	79	*	0.04	Granulocytes	2.68
Basophils	1255		0.67	Granulocytes	42.50
Eosinophils	889		0.48	Granulocytes	30.10
CD66b- Neutrophils	730		0.39	Granulocytes	24.72
Other	5634		N/A	All events	3.02

* Note, low count population (<100 events) will exhibit higher error and variation.

FLUIDIGM

Maxpar® Pathsetter™ Report

Powered by GemStone™

FCS File: AB2_1_1_1_cleaned.fcs

Sample 02 - HC

	Count	*	% Intact live	Parent	% Parent
Intact Live Cells	206916		100	All events	95.16
Lymphocytes	192965		93.26	Intact Live	93.26
CD3 T Cells	162395		78.48	Lymphocytes	84.16
CD8 T Cells	31691		15.32	CD3 T Cells	19.51
Naive	14905		7.20	CD8 T Cells	47.03
Central memory	3275		1.58	CD8 T Cells	10.33
Effector memory	6786		3.28	CD8 T Cells	21.41
Terminal effector	6725		3.25	CD8 T Cells	21.22
CD4 T Cells	124486		60.16	CD3 T Cells	76.66
Naive	68208		32.96	CD4 T Cells	54.79
Central memory	26743		12.92	CD4 T Cells	21.48
Effector memory	23828		11.52	CD4 T Cells	19.14
Terminal effector	5707		2.76	CD4 T Cells	4.58
Treg	3863		1.87	CD4 T cells	3.10
Th1-like	8927		4.31	CD4 T cells	7.17
Th2-like	13264		6.41	CD4 T cells	10.66
Th17-like	1292		0.62	CD4 T cells	1.04
Gamma Delta T Cells	6217		3.00	CD3 T Cells	3.83
MAIT & NKT CD4- Cells	1	*	0.00	CD3 T Cells	0.00
B Cells	11535		5.57	Lymphocytes	5.98
Naive	9021		4.36	B Cells	78.21
Memory	2482		1.20	B Cells	21.52
Plasmablasts	32	*	0.02	B Cells	0.28
NK Cells	19035		9.20	Lymphocytes	9.86
Early NK	6006		2.90	NK Cells	31.55
Late NK	13029		6.30	NK Cells	68.45
Monocytes	2698		1.30	Intact Live	1.30
Classical	1706		0.82	Monocytes	63.23
Transitional	269		0.13	Monocytes	9.97
Non-classical	723		0.35	Monocytes	26.80
Dendritic Cells	337		0.16	Intact Live	0.16
pDC	252		0.12	Dendritic Cells	74.78
mDC	85	*	0.04	Dendritic Cells	25.22
Granulocytes	902		0.44	Intact Live	0.44
Neutrophils	0	*	0	Granulocytes	0
Basophils	2	*	0.00	Granulocytes	0.22
Eosinophils	219		0.11	Granulocytes	24.28
CD66b- Neutrophils	681		0.33	Granulocytes	75.50
Other	10014		N/A	All events	4.84

* Note, low count population (<100 events) will exhibit higher error and variation.

FLUIDIGM

Maxpar® Pathsetter™ Report

Powered by GemStone™

FCS File: AB3_1_1_1_cleaned.fcs

Sample 03 - SLE

	Count	*	% Intact live	Parent	% Parent
Intact Live Cells	207127		100	All events	96.46
Lymphocytes	196778		95.00	Intact Live	95.00
CD3 T Cells	101938		49.22	Lymphocytes	51.80
CD8 T Cells	41996		20.28	CD3 T Cells	41.20
Naive	18496		8.93	CD8 T Cells	44.04
Central memory	1654		0.80	CD8 T Cells	3.94
Effector memory	7277		3.51	CD8 T Cells	17.33
Terminal effector	14569		7.03	CD8 T Cells	34.69
CD4 T Cells	57840		27.92	CD3 T Cells	56.74
Naive	25613		12.37	CD4 T Cells	44.28
Central memory	9151		4.42	CD4 T Cells	15.82
Effector memory	20396		9.85	CD4 T Cells	35.26
Terminal effector	2680		1.29	CD4 T Cells	4.63
Treg	6839		3.30	CD4 T cells	11.82
Th1-like	2855		1.38	CD4 T cells	4.94
Th2-like	9329		4.50	CD4 T cells	16.13
Th17-like	1315		0.63	CD4 T cells	2.27
Gamma Delta T Cells	1879		0.91	CD3 T Cells	1.84
MAIT & NKT CD4- Cells	223		0.11	CD3 T Cells	0.22
B Cells	91075		43.97	Lymphocytes	46.28
Naive	86480		41.75	B Cells	94.95
Memory	4560		2.20	B Cells	5.01
Plasmablasts	35	*	0.02	B Cells	0.04
NK Cells	3765		1.82	Lymphocytes	1.91
Early NK	1172		0.57	NK Cells	31.13
Late NK	2593		1.25	NK Cells	68.87
Monocytes	1542		0.74	Intact Live	0.74
Classical	1527		0.74	Monocytes	99.03
Transitional	11	*	0.01	Monocytes	0.71
Non-classical	4	*	0.00	Monocytes	0.26
Dendritic Cells	196		0.09	Intact Live	0.09
pDC	165		0.08	Dendritic Cells	84.18
mDC	31	*	0.01	Dendritic Cells	15.82
Granulocytes	1272		0.61	Intact Live	0.61
Neutrophils	0	*	0	Granulocytes	0
Basophils	3	*	0.00	Granulocytes	0.24
Eosinophils	103		0.05	Granulocytes	8.10
CD66b- Neutrophils	1166		0.56	Granulocytes	91.67
Other	7339		N/A	All events	3.54

* Note, low count population (<100 events) will exhibit higher error and variation.

FLUIDIGM

Maxpar® Pathsetter™ Report

Powered by GemStone™

FCS File: AB4_1_1_1_cleaned.fcs

Sample 04 - Case

	Count	*	% Intact live	Parent	% Parent
Intact Live Cells	167125		100	All events	95.93
Lymphocytes	132485		79.27	Intact Live	79.27
CD3 T Cells	100139		59.92	Lymphocytes	75.59
CD8 T Cells	35233		21.08	CD3 T Cells	35.18
Naive	21960		13.14	CD8 T Cells	62.33
Central memory	440		0.26	CD8 T Cells	1.25
Effector memory	4926		2.95	CD8 T Cells	13.98
Terminal effector	7907		4.73	CD8 T Cells	22.44
CD4 T Cells	62408		37.34	CD3 T Cells	62.32
Naive	39011		23.34	CD4 T Cells	62.51
Central memory	11615		6.95	CD4 T Cells	18.61
Effector memory	8783		5.26	CD4 T Cells	14.07
Terminal effector	2999		1.79	CD4 T Cells	4.81
Treg	2804		1.68	CD4 T cells	4.49
Th1-like	206		0.12	CD4 T cells	0.33
Th2-like	7007		4.19	CD4 T cells	11.23
Th17-like	131		0.08	CD4 T cells	0.21
Gamma Delta T Cells	2445		1.46	CD3 T Cells	2.44
MAIT & NKT CD4- Cells	53	*	0.03	CD3 T Cells	0.05
B Cells	5881		3.52	Lymphocytes	4.44
Naive	4774		2.86	B Cells	81.18
Memory	958		0.57	B Cells	16.29
Plasmablasts	149		0.09	B Cells	2.53
NK Cells	26465		15.84	Lymphocytes	19.98
Early NK	8321		4.98	NK Cells	31.44
Late NK	18144		10.86	NK Cells	68.56
Monocytes	14201		8.50	Intact Live	8.50
Classical	11116		6.65	Monocytes	78.28
Transitional	2126		1.27	Monocytes	14.97
Non-classical	959		0.57	Monocytes	6.75
Dendritic Cells	1143		0.68	Intact Live	0.68
pDC	440		0.26	Dendritic Cells	38.50
mDC	703		0.42	Dendritic Cells	61.50
Granulocytes	12493		7.48	Intact Live	7.48
Neutrophils	3149		1.88	Granulocytes	25.21
Basophils	56	*	0.03	Granulocytes	0.45
Eosinophils	8657		5.18	Granulocytes	69.29
CD66b- Neutrophils	631		0.38	Granulocytes	5.05
Other	6803		N/A	All events	4.07

* Note, low count population (<100 events) will exhibit higher error and variation.

FLUIDIGM

Maxpar® Pathsetter™ Report

Powered by GemStone™

FCS File: AB5_1_1_1_cleaned.fcs

Sample 05 - HC

	Count	*	% Intact live	Parent	% Parent
Intact Live Cells	200619		100	All events	93.86
Lymphocytes	181010		90.23	Intact Live	90.23
CD3 T Cells	158308		78.91	Lymphocytes	87.46
CD8 T Cells	57961		28.89	CD3 T Cells	36.61
Naive	18045		8.99	CD8 T Cells	31.13
Central memory	2125		1.06	CD8 T Cells	3.67
Effector memory	14149		7.05	CD8 T Cells	24.41
Terminal effector	23642		11.78	CD8 T Cells	40.79
CD4 T Cells	93445		46.58	CD3 T Cells	59.03
Naive	37265		18.58	CD4 T Cells	39.88
Central memory	10246		5.11	CD4 T Cells	10.96
Effector memory	17911		8.93	CD4 T Cells	19.17
Terminal effector	28023		13.97	CD4 T Cells	29.99
Treg	3878		1.93	CD4 T cells	4.15
Th1-like	24935		12.43	CD4 T cells	26.68
Th2-like	3807		1.90	CD4 T cells	4.07
Th17-like	2873		1.43	CD4 T cells	3.07
Gamma Delta T Cells	5344		2.66	CD3 T Cells	3.38
MAIT & NKT CD4- Cells	1558		0.78	CD3 T Cells	0.98
B Cells	16929		8.44	Lymphocytes	9.35
Naive	8049		4.01	B Cells	47.55
Memory	8677		4.33	B Cells	51.26
Plasmablasts	203		0.10	B Cells	1.20
NK Cells	5773		2.88	Lymphocytes	3.19
Early NK	1815		0.90	NK Cells	31.44
Late NK	3958		1.97	NK Cells	68.56
Monocytes	6034		3.01	Intact Live	3.01
Classical	3945		1.97	Monocytes	65.38
Transitional	572		0.29	Monocytes	9.48
Non-classical	1517		0.76	Monocytes	25.14
Dendritic Cells	528		0.26	Intact Live	0.26
pDC	377		0.19	Dendritic Cells	71.40
mDC	151		0.08	Dendritic Cells	28.60
Granulocytes	725		0.36	Intact Live	0.36
Neutrophils	4	*	0.00	Granulocytes	0.55
Basophils	389		0.19	Granulocytes	53.66
Eosinophils	215		0.11	Granulocytes	29.66
CD66b- Neutrophils	117		0.06	Granulocytes	16.14
Other	12322		N/A	All events	6.14

* Note, low count population (<100 events) will exhibit higher error and variation.

FLUIDIGM

Maxpar® Pathsetter™ Report

Powered by GemStone™

FCS File: AB6_1_1_cleaned.fcs

Sample 06 - HC

	Count	*	% Intact live	Parent	% Parent
Intact Live Cells	204936		100	All events	96.29
Lymphocytes	186899		91.20	Intact Live	91.20
CD3 T Cells	122011		59.54	Lymphocytes	65.28
CD8 T Cells	32264		15.74	CD3 T Cells	26.44
Naive	21553		10.52	CD8 T Cells	66.80
Central memory	882		0.43	CD8 T Cells	2.73
Effector memory	8946		4.37	CD8 T Cells	27.73
Terminal effector	883		0.43	CD8 T Cells	2.74
CD4 T Cells	81073		39.56	CD3 T Cells	66.45
Naive	47614		23.23	CD4 T Cells	58.73
Central memory	9557		4.66	CD4 T Cells	11.79
Effector memory	19665		9.60	CD4 T Cells	24.26
Terminal effector	4237		2.07	CD4 T Cells	5.23
Treg	4355		2.13	CD4 T cells	5.37
Th1-like	4464		2.18	CD4 T cells	5.51
Th2-like	8726		4.26	CD4 T cells	10.76
Th17-like	2789		1.36	CD4 T cells	3.44
Gamma Delta T Cells	6668		3.25	CD3 T Cells	5.47
MAIT & NKT CD4- Cells	2006		0.98	CD3 T Cells	1.64
B Cells	24369		11.89	Lymphocytes	13.04
Naive	20396		9.95	B Cells	83.70
Memory	3886		1.90	B Cells	15.95
Plasmablasts	87	*	0.04	B Cells	0.36
NK Cells	40519		19.77	Lymphocytes	21.68
Early NK	13078		6.38	NK Cells	32.28
Late NK	27441		13.39	NK Cells	67.72
Monocytes	6902		3.37	Intact Live	3.37
Classical	4792		2.34	Monocytes	69.43
Transitional	498		0.24	Monocytes	7.22
Non-classical	1612		0.79	Monocytes	23.36
Dendritic Cells	2557		1.25	Intact Live	1.25
pDC	637		0.31	Dendritic Cells	24.91
mDC	1920		0.94	Dendritic Cells	75.09
Granulocytes	984		0.48	Intact Live	0.48
Neutrophils	58	*	0.03	Granulocytes	5.89
Basophils	307		0.15	Granulocytes	31.20
Eosinophils	324		0.16	Granulocytes	32.93
CD66b- Neutrophils	295		0.14	Granulocytes	29.98
Other	7594		N/A	All events	3.71

* Note, low count population (<100 events) will exhibit higher error and variation.

FLUIDIGM

Maxpar® Pathsetter™ Report

Powered by GemStone™

FCS File: AB7_1_1_cleaned.fcs

Sample 07 - HC

	Count	*	% Intact live	Parent	% Parent
Intact Live Cells	111624		100	All events	93.18
Lymphocytes	93001		83.32	Intact Live	83.32
CD3 T Cells	76492		68.53	Lymphocytes	82.25
CD8 T Cells	32487		29.10	CD3 T Cells	42.47
Naive	15991		14.33	CD8 T Cells	49.22
Central memory	694		0.62	CD8 T Cells	2.14
Effector memory	10606		9.50	CD8 T Cells	32.65
Terminal effector	5196		4.65	CD8 T Cells	15.99
CD4 T Cells	35200		31.53	CD3 T Cells	46.02
Naive	11578		10.37	CD4 T Cells	32.89
Central memory	4732		4.24	CD4 T Cells	13.44
Effector memory	14792		13.25	CD4 T Cells	42.02
Terminal effector	4098		3.67	CD4 T Cells	11.64
Treg	2429		2.18	CD4 T cells	6.90
Th1-like	4507		4.04	CD4 T cells	12.80
Th2-like	5214		4.67	CD4 T cells	14.81
Th17-like	1550		1.39	CD4 T cells	4.40
Gamma Delta T Cells	4699		4.21	CD3 T Cells	6.14
MAIT & NKT CD4- Cells	4106		3.68	CD3 T Cells	5.37
B Cells	10515		9.42	Lymphocytes	11.31
Naive	5892		5.28	B Cells	56.03
Memory	4517		4.05	B Cells	42.96
Plasmablasts	106		0.09	B Cells	1.01
NK Cells	5994		5.37	Lymphocytes	6.45
Early NK	1898		1.70	NK Cells	31.66
Late NK	4096		3.67	NK Cells	68.34
Monocytes	9132		8.18	Intact Live	8.18
Classical	5884		5.27	Monocytes	64.43
Transitional	1562		1.40	Monocytes	17.10
Non-classical	1686		1.51	Monocytes	18.46
Dendritic Cells	1535		1.38	Intact Live	1.38
pDC	1007		0.90	Dendritic Cells	65.60
mDC	528		0.47	Dendritic Cells	34.40
Granulocytes	344		0.31	Intact Live	0.31
Neutrophils	0	*	0	Granulocytes	0
Basophils	22	*	0.02	Granulocytes	6.40
Eosinophils	206		0.18	Granulocytes	59.88
CD66b- Neutrophils	116		0.10	Granulocytes	33.72
Other	7612		N/A	All events	6.82

* Note, low count population (<100 events) will exhibit higher error and variation.

FLUIDIGM

Maxpar® Pathsetter™ Report

Powered by GemStone™

FCS File: AB8_1_1_1_cleaned.fcs

Sample 08 - SLE

	Count	*	% Intact live	Parent	% Parent
Intact Live Cells	11403		100	All events	94.41
Lymphocytes	10532		92.36	Intact Live	92.36
CD3 T Cells	10357		90.83	Lymphocytes	98.34
CD8 T Cells	2588		22.70	CD3 T Cells	24.99
Naive	329		2.89	CD8 T Cells	12.71
Central memory	40	*	0.35	CD8 T Cells	1.55
Effector memory	647		5.67	CD8 T Cells	25
Terminal effector	1572		13.79	CD8 T Cells	60.74
CD4 T Cells	6570		57.62	CD3 T Cells	63.44
Naive	1933		16.95	CD4 T Cells	29.42
Central memory	782		6.86	CD4 T Cells	11.90
Effector memory	2005		17.58	CD4 T Cells	30.52
Terminal effector	1850		16.22	CD4 T Cells	28.16
Treg	468		4.10	CD4 T cells	7.12
Th1-like	740		6.49	CD4 T cells	11.26
Th2-like	931		8.16	CD4 T cells	14.17
Th17-like	143		1.25	CD4 T cells	2.18
Gamma Delta T Cells	1180		10.35	CD3 T Cells	11.39
MAIT & NKT CD4- Cells	19	*	0.17	CD3 T Cells	0.18
B Cells	24	*	0.21	Lymphocytes	0.23
Naive	3	*	0.03	B Cells	12.50
Memory	21	*	0.18	B Cells	87.50
Plasmablasts	0	*	0	B Cells	0
NK Cells	151		1.32	Lymphocytes	1.43
Early NK	49	*	0.43	NK Cells	32.45
Late NK	102		0.89	NK Cells	67.55
Monocytes	163		1.43	Intact Live	1.43
Classical	136		1.19	Monocytes	83.44
Transitional	18	*	0.16	Monocytes	11.04
Non-classical	9	*	0.08	Monocytes	5.52
Dendritic Cells	39	*	0.34	Intact Live	0.34
pDC	9	*	0.08	Dendritic Cells	23.08
mDC	30	*	0.26	Dendritic Cells	76.92
Granulocytes	32	*	0.28	Intact Live	0.28
Neutrophils	0	*	0	Granulocytes	0
Basophils	2	*	0.02	Granulocytes	6.25
Eosinophils	3		0.03	Granulocytes	9.38
CD66b- Neutrophils	27		0.24	Granulocytes	84.38
Other	637		N/A	All events	5.59

* Note, low count population (<100 events) will exhibit higher error and variation.
