# Supplementary figures and images for: Increased interferon I signaling, DNA damage response and evidence of T-cell exhaustion in a patient with combined interferonopathy (Aicardi-Goutières Syndrome, AGS) and cohesinopathy (Cornelia de Lange Syndrome, CdLS)
Source: Pediatr Rheumatol Online J. 2025 Jan 27;23:11. doi: 10.1186/s12969-024-01050-7 (PMC11770959; doi:10.1186/s12969-024-01050-7)

Supplementary Figure 1

A

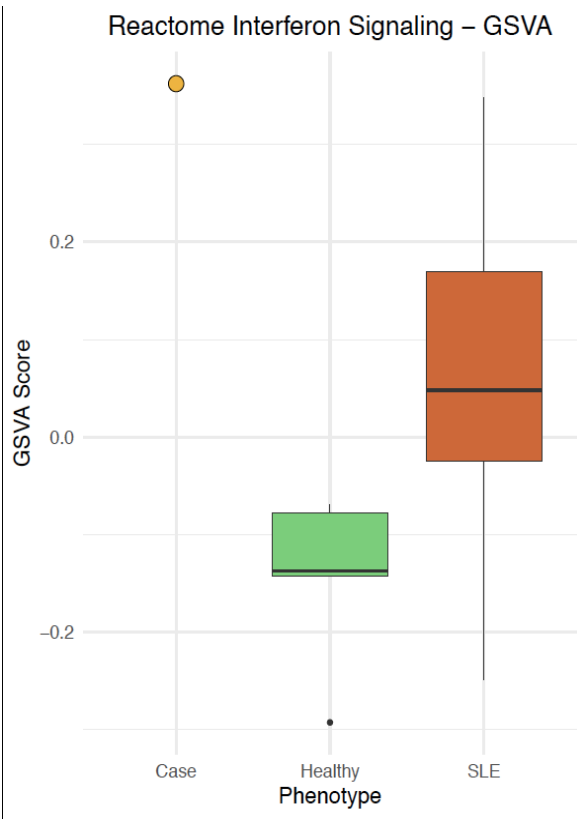

B

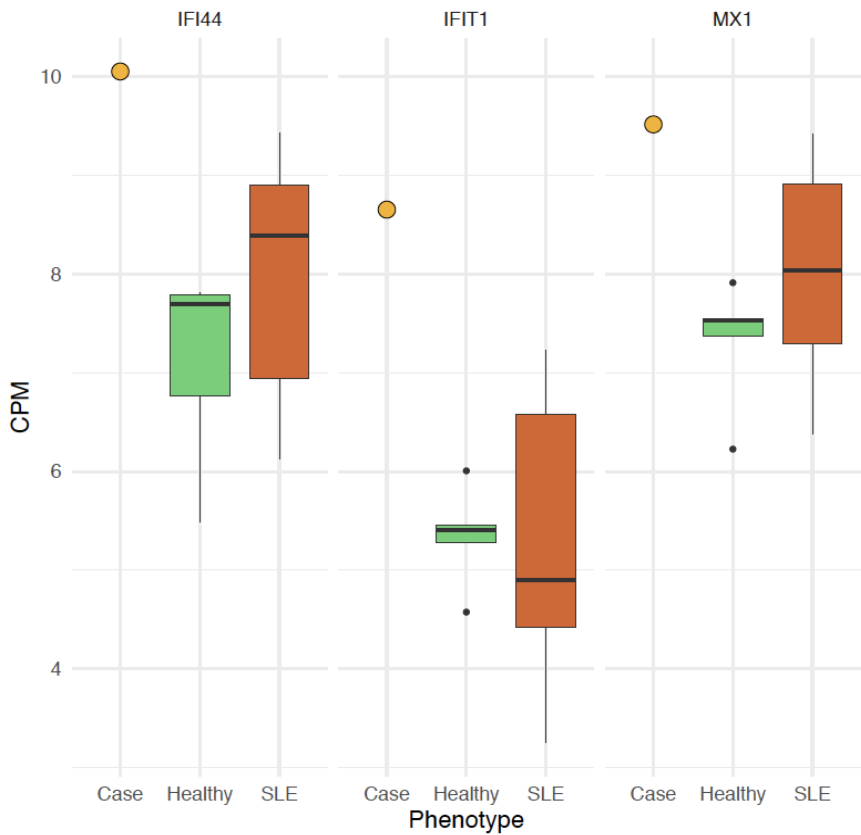

C

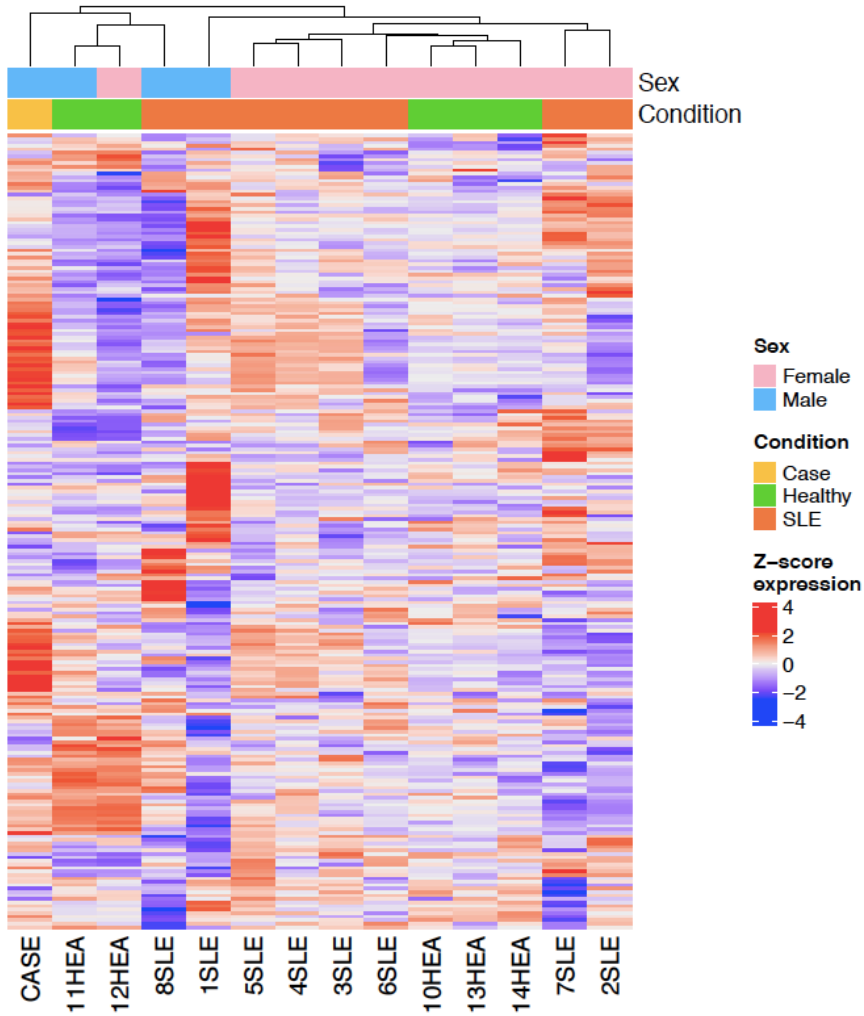

Supplement: Supplementary file 5 — Supplementary Material 5: Supplementary Figure 1. A. Cumulative IFN-score (Reactome - IFN signaling) for Case, SLE patients and healthy controls. B. Gene-specific expression of genes utilized for IFN-score in Case, SLE patients and healthy controls. C. Heatmap of the expression of IFN-related genes in Case, SLE patients and healthy controls. [file 12969_2024_1050_MOESM5_ESM.pdf]

Supplementary Figure 2

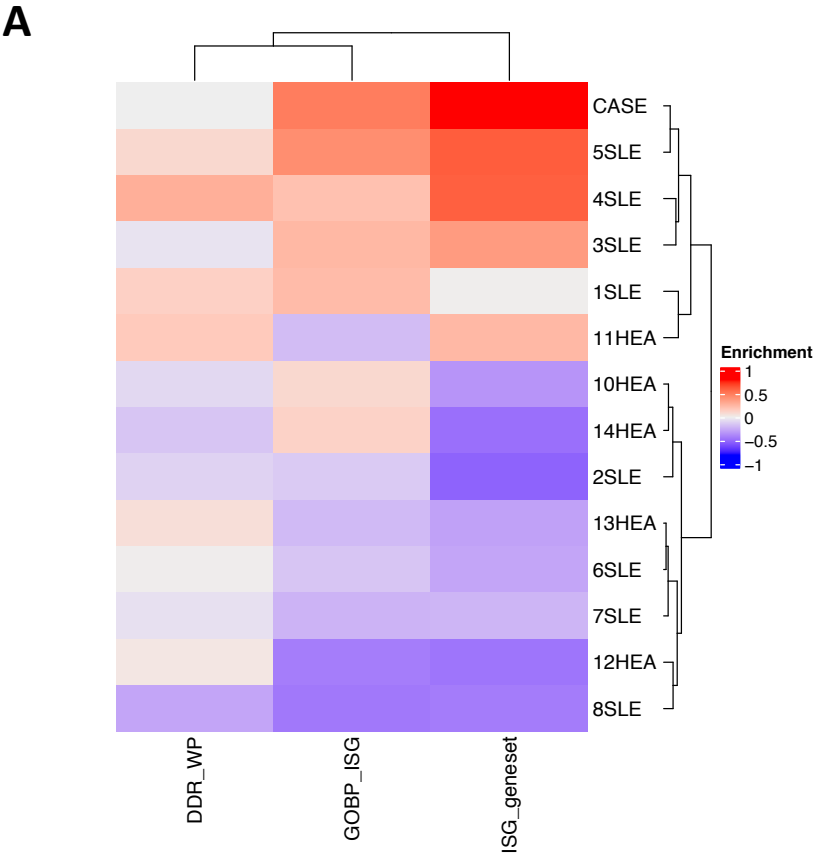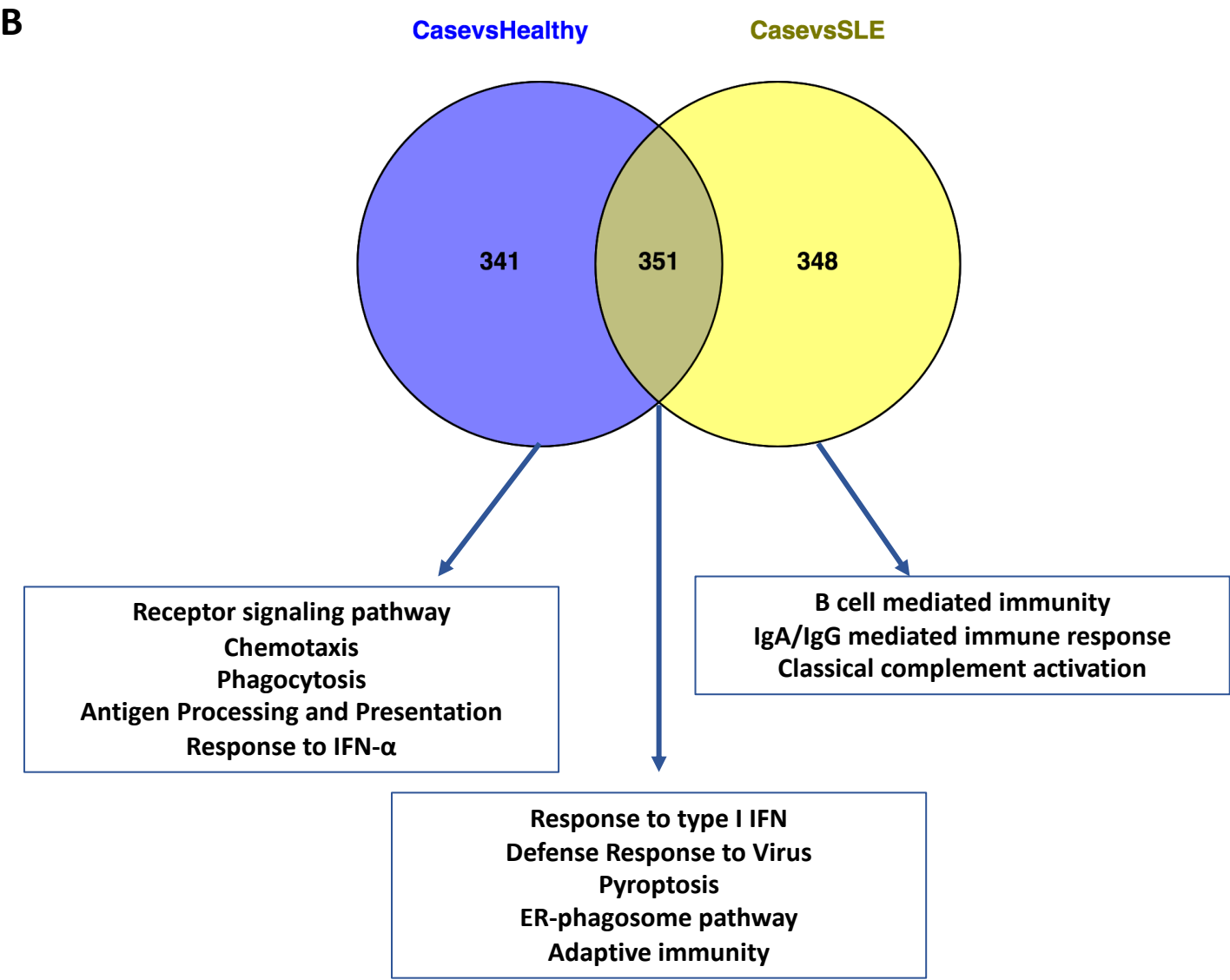

Supplement: Supplementary file 6 — Supplementary Material 6: Supplementary Figure 2. A. Heatmap showing the enrichment in WikiPathways DNA Damage Response, GOBP: Interferon Mediated Signaling Pathway and the publication-derived Interferon Stimulated Genes signature, as calculated by GSVA. B. Venn diagram of differentially expressed genes between 1) CasevsHealthy and 2) CasevsSLE. [file 12969_2024_1050_MOESM6_ESM.pdf]

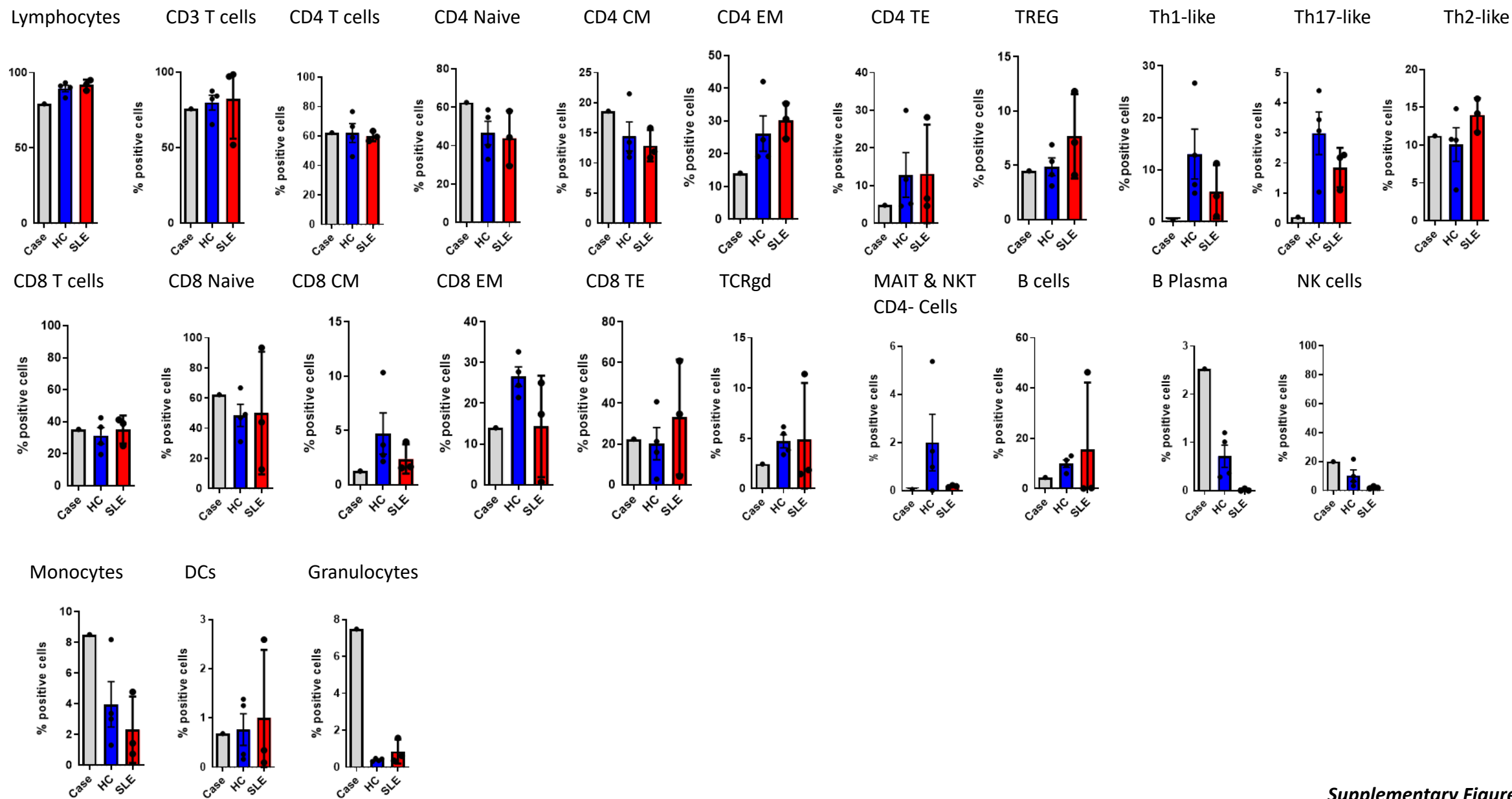

**Supplementary Figure 3**

Supplement: Supplementary file 7 — Supplementary Material 7: Supplementrary Figure 3. Automated Immunophenotyping analysis of PBMCs from the Case, SLE patients and healthy controls with the Maxpar Pathsetter workflow. Boxplots showing relative frequency (% positive cells of live singlet PBMCs) of the identified cell types from the automated workflow for the three conditions, HC, SLE and the Case (CM, Central Memory, EM, Effector Memory, TE, Terminal Effector, TREG, T regulatory). [file 12969_2024_1050_MOESM7_ESM.pdf]

Supplementary Figure 4

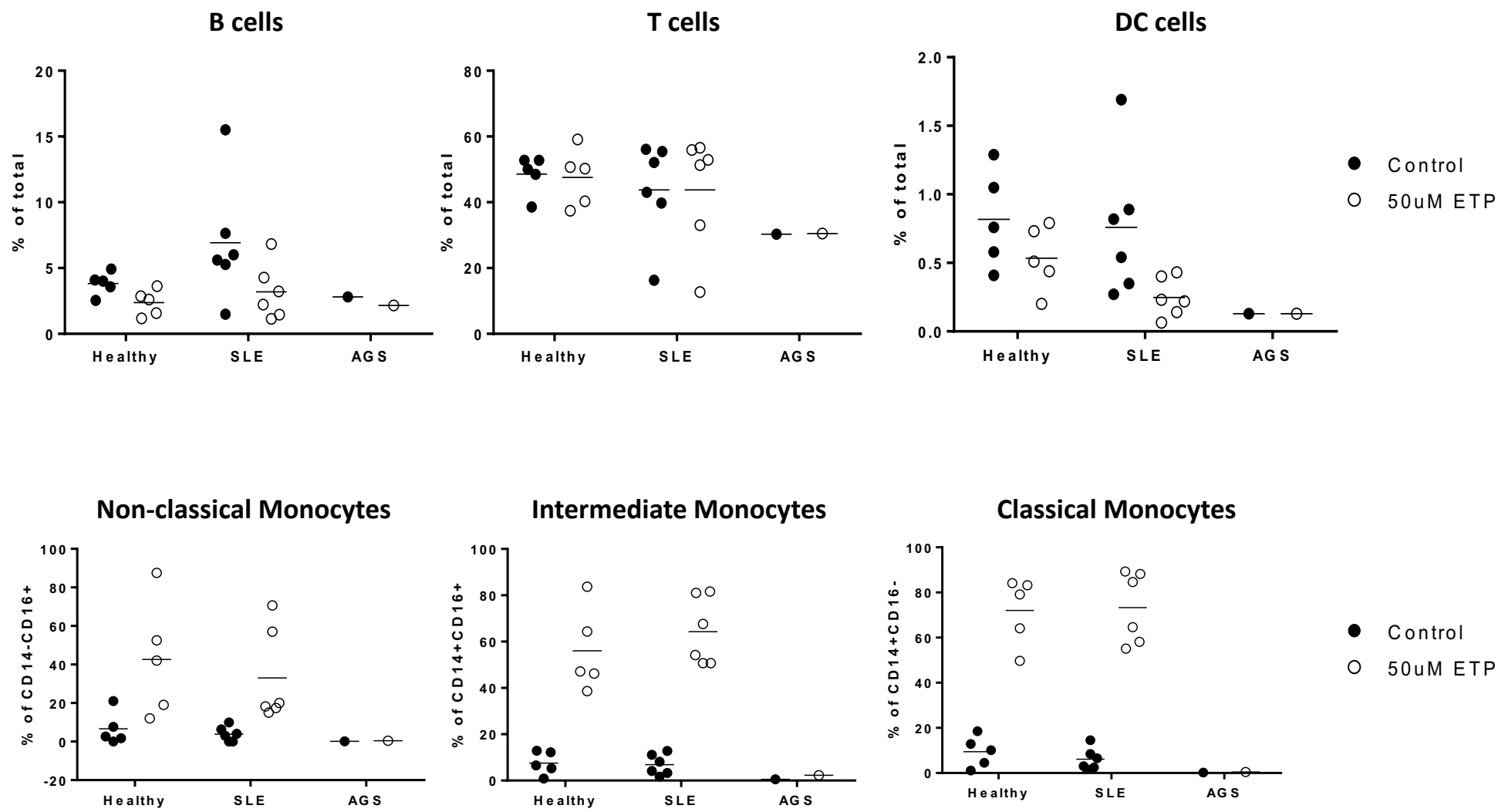

Supplement: Supplementary file 8 — Supplementary Material 8: Supplementary Figure 4. Percentage of different immune cell types (B cells, T cells, Dendritic Cells, classical monocytes, non-classical monocytes, intermediate monocytes) with high abundance of phospho-γH2AX and/or phospho-IRF3 following 16h of culture +/- 50μM Etoposide (ETP). [file 12969_2024_1050_MOESM8_ESM.pdf]
